# Inflammation as a Driver of Clonal Evolution in Myeloproliferative Neoplasm

**DOI:** 10.1155/2015/606819

**Published:** 2015-10-11

**Authors:** Angela G. Fleischman

**Affiliations:** University of California, Irvine, CA 92697, USA

## Abstract

Our understanding of inflammation's role in the pathogenesis of myeloproliferative neoplasm (MPN) is evolving. The impact of chronic inflammation, a characteristic feature of MPN, likely goes far beyond its role as a driver of constitutional symptoms. An inflammatory response to the neoplastic clone may be responsible for some pathologic aspects of MPN. Moreover, *JAK2V617F* mutated hematopoietic stem and progenitor cells are resistant to inflammation, and this gives the neoplastic clone a selective advantage allowing for its clonal expansion. Because inflammation plays a central role in MPN inflammation is a logical therapeutic target in MPN.

## 1. Introduction

Inflammation has a central role in myeloproliferative neoplasm (MPN). Excessive inflammation not only causes many of the debilitating symptoms associated with the disease but also drives the selective expansion of the neoplastic clone [1–3]. Inflammation may also be directly responsible for some pathologic features of the disease such as bone marrow fibrosis and anemia [4–9]. Below, I will provide evidence for increased inflammatory cytokine production in MPN, describe the effect of inflammation on both normal and neoplastic hematopoietic stem and progenitor cells, and propose a model whereby inflammatory insult upon a vulnerable hematopoietic stem cell pool drives the emergence of the MPN neoplastic clone.

## 2. What Causes Inflammation in MPN?

A deranged inflammatory cytokine profile is a shared characteristic of both humans with MPN as well as mouse MPN models. This suggests that the* JAK2V617F* clone is responsible for inflammation but exactly how the* JAK2V617F* clone induces inflammation is unclear. Excessive inflammatory cytokine production is not exclusive to the neoplastic cells in MPN. For example, the inflammatory cytokine tumor necrosis factor-alpha (TNF) is equally overproduced in both the* JAK2V617F* and the* JAK2WT* cells from MPN patients and in MPN mice (AF unpublished results). Moreover, mice injected with Ba/F3 cells expressing* JAK2V617F* have elevated production of inflammatory cytokines including TNF and IL-6, but the Ba/F3* JAK2V617F* cells are not the source of these cytokines [10]. Ross Levine's group used single cell cytokine analysis to measure cytokine production in a mouse MPN model [11] and found that both neoplastic and nonneoplastic cells produce excessive inflammatory cytokines. They also used STAT3 knockout mice to investigate the role of STAT3 mediated cytokine production in the pathogenesis of MPN. In mice with pan-hematopoietic deletion of STAT3, the MPN phenotype was attenuated in a* MPLW515L* MPN model, but if STAT3 was deleted in the neoplastic cells but remained intact in the nonneoplastic cells, the disease phenotype remained robust [11]. Taken together, these data suggest that the MPN neoplastic clone may induce an inflammatory reaction by nonneoplastic host cells and that the inflammation mediated by these nonneoplastic cells plays a key role in MPN pathogenesis.

Inflammatory cells of both the innate and adaptive immune systems contribute to MPN pathology in mouse models, supporting the notion that some pathologic features of MPN are caused by the host's reaction to the neoplastic clone rather than a direct consequence of the neoplastic clone itself. For example, erythrocytosis was attenuated in MPN mouse models deficient in macrophages [12, 13]. We have found that in a background devoid of T and B cells (RAG2^−/−^) a* JAK2V617F* transduction-transplantation MPN model had no fibrosis, attenuated splenomegaly, and reduced leukocytosis yet erythrocytosis and excessive megakaryopoiesis were preserved (AF, unpublished results). These data demonstrate that each cellular subset of the immune system affects distinct aspects of MPN pathophysiology.

**Figure 1:**
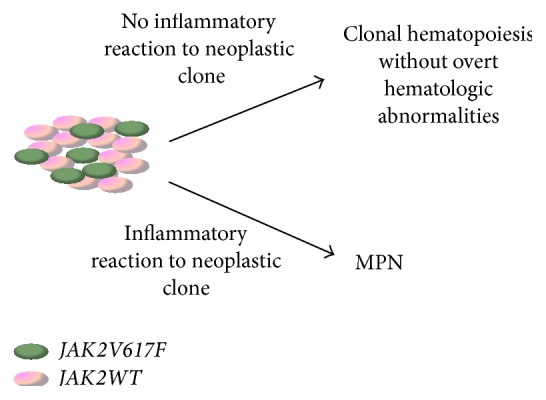
Putative role of inflammation in shaping MPN disease phenotype.

The nature or extent of the host's inflammatory response to a* JAK2V617F* clone could modulate the MPN phenotype (Figure 1). It is possible that an inflammatory response to the* JAK2V617F* clone is required to develop a clinically relevant MPN, and without this inflammatory response* JAK2V617F* results in clonal hematopoiesis without overt hematologic abnormalities. This would explain the observation of normal elderly individuals with a detectable* JAK2V617F* clone [14–16]. It is also possible that the nature of the host's inflammatory response to the clone shapes the resulting MPN phenotype that may help explain why this one mutation can lead to three distinct disease entities.

## 3. Inflammation as a Therapeutic Target in MPN

Targeting inflammation is a logical therapeutic approach in MPN. JAK inhibitors are not only used in MPN but are also utilized in autoimmune and inflammatory diseases [17] because JAK/STAT signalling is involved in the production of many inflammatory cytokines. Treatment of MPN patients with the JAK1/2 inhibitor ruxolitinib results in prompt resolution of constitutional symptoms concurrent with reduction in the inflammatory cytokines profile in plasma [18]. Ruxolitinib does not reduce* JAK2V617F* allele burden immediately, suggesting that its effect is not by direct targeting of the* JAK2V617F* neoplastic cells. Over time, however, there appears to be a decrease in the* JAK2V617F* allele burden [19] in patients treated with ruxolitinib.

It is possible that ruxolitinib's effect in MPN is via its anti-inflammatory properties and not necessarily through its inhibition of the mutant JAK2. Decreasing inflammation may reduce the selective advantage of the* JAK2V617F* clone and lead to its contraction, albeit slow. Inhibitors with primarily JAK1 inhibitory activity and little JAK2 inhibitory activity have been investigated in myelofibrosis (MF) to test this hypothesis [20]. Treatment of MF patients with a JAK1 inhibitor resulted in improvements in MF-related symptoms and a modest decrease in spleen size (abstract 714 ASH 2014) demonstrating that agents that selectively target JAK1 while sparing JAK2 do have activity in MF.

Perhaps chronic inflammation produces an environment that is highly selective for hematopoietic stem cell clones with MPN associated mutations such as* JAK2V617F*. How exactly MPN associated mutations may give hematopoietic stem cells a selective advantage in the setting of chronic inflammation is unknown, but the known consequences of chronic inflammation on hematopoietic stem cells provide us with some clues.

## 4. Impact of Chronic Inflammation on Hematopoietic Stem Cells

Chronic inflammation causes age-related hematopoietic stem cell (HSC) functional decline. The ability of hematopoietic stem cells to be quickly called into cycle is crucial for the rapid hematopoiesis required during times of stress, but HSC bombarded by chronic inflammation eventually exhausts. This HSC exhaustion is mediated by chronic cycling of HSC. In mouse model systems repeated exposures to inflammatory stimuli such as lipopolysaccharide (LPS) [21] negatively affected HSC. HSC function was also compromised in mice with a deletion of miR-146a, a micro-RNA whose role is to suppress inflammatory cytokine production. Aged miR-146a knock-out mice developed hematopoietic stem cell exhaustion, myeloproliferation, marrow fibrosis, and extramedullary hematopoiesis. This stem cell exhaustion and myeloproliferative phenotype could be induced in young miR-146a^−/−^ mice by chronic bacterial exposure. This suggests that exposure to pathogens and the ensuing inflammatory response catalyzes HSC exhaustion. To further illustrate the role of inflammation in HSC exhaustion, a cross of the miR146a^−/−^ mice onto a RAG1^−/−^ background (which lacks T and B cells) rescued HSC from exhaustion. Together these data demonstrate that chronic inflammation leads to HSC exhaustion and promotes the development of myeloproliferation, marrow fibrosis, and extramedullary hematopoiesis.

## 5. Thrombopoietin/MPL Signaling Pathway in HSC Quiescence and Its Relevance to MPN

The thrombopoietin (TPO) signaling axis plays a central role in hematopoietic stem cell cycling [22]. The hematopoietic stem cell phenotype in mice deficient in TPO and its receptor MPL highlight the importance of this pathway for HSC maintenance. MPL signaling maintains HSC quiescence. HSC from TPO^−/−^ mice display accelerated cell-cycle kinetics and reduced transcriptional expression of negative cell-cycle regulators. This chronic cycling leads to HSC attrition, resulting in a 150-fold reduction of HSC in TPO^−/−^ mice. Mutations which enhance MPL signaling result in superior hematopoietic stem cell reconstitution ability and self-renewal in mouse models. This includes mice deficient in negative regulators of MPL signaling such as Lnk [23] and MERIT-40 [24].

Activation of the MPL pathway is the common theme among mutations associated with MPN including* MPL*,* JAK2*, and* LNK*. It is currently unknown whether calreticulin mutations affect the MPL signaling pathway, but clues such as high expression of mutated calreticulin in MPN megakaryocytes [25] implicate a potential role for calreticulin in megakaryopoiesis. The observation that thrombopoietin mimetics induce reversible marrow fibrosis [26] demonstrates that excessive signaling through MPL is in itself capable of mediating bone marrow fibrosis.

Intact MPL signaling is required for development of* JAK2V617F* induced MPN in mouse models [27]. Hitchcock's group crossed* JAK2V617F* transgenic mice onto a* MPL* ^−/−^ background which resulted in thrombocytopenia and lessened neutrophilia compared to* JAK2V617F* transgenic mice on a* MPL* wild-type background. Crossing of* JAK2V617F* transgenic mice onto a thrombopoietin deficient (*TPO* ^−/−^) background however had a different effect.* TPO* ^−/−^ mice are thrombocytopenic, and expression of* JAK2V617F* restored platelets to a normal level.* JAK2V617F* mice on a* TPO* ^−/−^ background had larger spleens and increased numbers of megakaryocytes in the spleen compared to* JAK2V617F* transgenics on a wild-type background.

Spivak's group also found that a functional thrombopoietin receptor is required for development of polycythemia vera in a mouse transgenic model of PV (abstract 427, ASH 2012). In their hands, abrogation of TPO production (*TPO* ^−/−^) delayed erythrocytosis, decreased spleen size, and reversed fibrosis. They found that loss of functional MPL ameliorated PV, with a decreased hematocrit, decreased leukocytosis, decreased platelet count, and reversal of fibrosis. Taken together, these data demonstrate that activation of the Tpo/MPL signaling pathway is crucial for the MPN disease phenotype. It is also attempting to speculate that activation of the MPL signaling pathway by* JAK2V617F* may also affect HSC cycling.

## 6. *JAK2V617F* Downregulates MPL

Decreased expression of MPL on the cell surface of platelets, megakaryocytes, and CD34^+^ cells is an established feature of polycythemia vera and myelofibrosis [28–30].* JAK2V617F* directly downmodulates MPL [31]. Wild-type* JAK2* but not* JAK2V617F* renders MPL resistant to endoglycosidase H-mediated degradation which allows MPL to be recycled back to the cell surface after ligand binding. JAK2 wild-type cells will have high levels of MPL on their cell surface when exposed to high concentrations of TPO, leading to quiescence and apoptosis. In the context of* JAK2V617F*, MPL is internalized and ubiquitinated and undergoes proteasomal degradation. As a result, cell surface expression of MPL is much lower in cells with* JAK2V617F*, facilitating resistance to the quiescence/apoptosis that would normally be encountered after exposure to high levels of TPO and allowing these cells to continue to proliferate.

## 7. Reactive Oxygen Species as Mediators of DNA Damage and HSC Aging

HSC cycling induces ROS which results in DNA damage, and this accumulation of DNA damage leads to HSC aging [32]. Genetic defects that cause increased production of ROS result in premature HSC aging and are associated with bone marrow failure syndromes such as Fanconi Anemia (FA). In a mouse model of FA the bone marrow failure phenotype only becomes evident when mice are exposed to chronic inflammation [32]. This inflammation induced HSC exhaustion can be prevented in FA mice with the ROS scavenger N-acetyl cysteine (NAC) [33]. This demonstrates that chronic inflammation promotes HSC attrition by increasing ROS production and more importantly that protection of HSC from inflammatory stress may be of value therapeutically for those individuals susceptible to accelerated HSC aging.

Increased ROS is a feature of MPN patients and mouse models.* JAK2V617F* cells confer paracrine DNA damage to neighboring normal cells as well as to themselves through increased ROS. The increased ROS was found to be mediated by lipocalin-2 (Lcn2) which is overexpressed in* JAK2V617F* cells [34, 35]. Normal bystander hematopoietic cells exposed to Lcn2 demonstrated p53 pathway activation, increased apoptosis, and decreased cellular proliferation.* JAK2V617F* cells were resistant to Lcn2-induced growth suppression which conferred a relative growth advantage to* JAK2V617F* clones. Treatment of a mouse knock-in* JAK2V617F* model with NAC reduced erythrocytosis and splenomegaly [36] suggesting that accumulation of ROS may play a direct role in the pathogenesis of MPN and that therapeutics that reduce ROS could be of value in MPN.

## 8. *JAK2V617F* Cells Have a Selective Advantage in Inflammatory Environments

**Figure 2:**
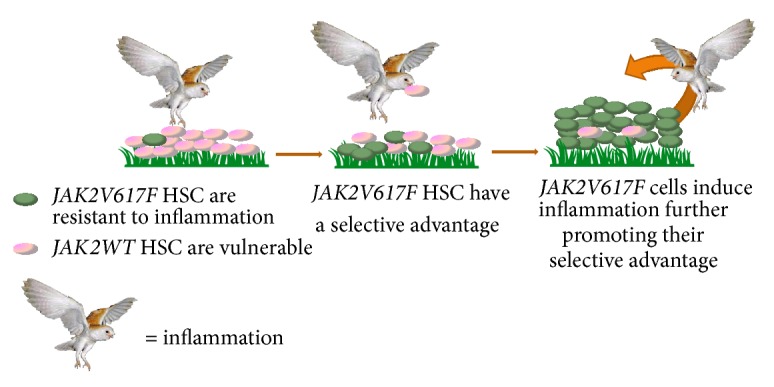
Model of inflammation as a driver of expansion of the* JAK2V617F* clone in MPN.* JAK2V617F*-mutated hematopoietic stem cells (HSC) are resistant to inflammation giving them a selective advantage in environments with high inflammation.

Chronic inflammation may promote the emergence of the* JAK2V617F* neoplastic clone because* JAK2V617F* mutated hematopoietic progenitor cells are resistant to inflammatory cues which suppress normal hematopoietic progenitors (Figure 2). MPN hematopoietic cells overproduce inflammatory cytokines including tumor necrosis factor-alpha (TNF). We have found that the* JAK2V617F* mutation confers upon hematopoietic progenitor cells high-level resistance to the suppressive effects of TNF [1]. Consequently, because the overproduction of TNF suppresses the replicative activity of the nonmutated progenitors while enhancing the replication of the* JAK2V617F* mutant progenitors, the overall influence of the mutation* in vivo* is to alter the coefficient of selection in favor of the* JAK2V617F* clone.

Neoplastic cells in MPN have been found to be resistant to other specific inflammatory cytokines. This includes IL-33, a member of the IL-1 family of cytokines, which has been implicated in many autoimmune diseases [37]. Transplantation of bone marrow from* JAK2V617F* transgenic mice into lethally irradiated IL-33^−/−^ mice delayed the onset of MPN, demonstrating that IL-33 production by stromal cells promotes development of MPN. Increased IL-33-expressing cells were detected in bone marrow biopsies from MPN patients. Exogenous IL-33 promoted colony formation by primary CD34^+^ MPN stem/progenitor cells from patients and also improved the survival of* JAK2V617F* positive cell lines [38]. Together, these data demonstrate that IL-33 promotes the growth of* JAK2V617F* mutated cells.

## 9. Does Inflammation Precede the* JAK2V617F* Clone?

It is clear that concomitant inflammation supports MPN pathogenesis, but it is also possible that inflammation precedes the development of MPN and may be a predisposing factor to acquire MPN. A prior history of any autoimmune disease has been found to be associated with a significantly increased risk of MPN. Specifically, there is an increased risk of MPN with prior immune thrombocytopenia (OR = 2.9), Crohn's disease (1.8), polymyalgia rheumatic (1.7), giant cell arteritis (5.9), Reiter's syndrome (15.9), and aplastic anemia (7.8) [39]. Each of these autoimmune diseases is associated with substantial overproduction of inflammatory cytokines, including TNF [40, 41].

We are working to determine whether exaggerated production of inflammatory cytokines in response to immunogenic stimuli may be a predisposing factor to acquire MPN. We find that monocytes from MPN patients produce excessive amounts of TNF after stimulation through Toll-like receptors (TLR) and crucial pattern recognition receptors for microbial products (Abstract 4584, ASH 2014 annual meeting). This excessive production of TNF is due to a defect in the negative regulatory feedback loop which normally serves to dampen TNF production. Both mutant and nonmutant monocytes from MPN patients overproduce TNF, demonstrating the excessive cytokine production is not directly mediated by* JAK2V617F*.

## 10. Conclusion

MPN is a unique hematologic malignancy with a multifaceted and complex pathobiology. The unrestrained expansion of mature myeloid cells can be directly attributed to the constitutive activation of JAK2 by* JAK2V617F* but many of the other pathologic features of the disease such as marrow fibrosis, constitutional symptoms, and cytopenias could be due to the host's inflammatory reaction to the neoplastic clone. It is clear that inflammation plays a critical role in the pathogenesis of MPN but the details are still evolving. A more in-depth understanding of why inflammation is high in MPN and how the host's immune system responds to the neoplastic clone will likely reveal fundamental insights into MPN disease pathogenesis and will also identify novel therapeutic targets in this disease.
